# The mediating role of psychological capital on the relationship between perceived stress and self-directed learning ability in nursing students

**DOI:** 10.1186/s12912-024-02094-6

**Published:** 2024-06-17

**Authors:** Dan Yang, Wenkai Zheng, Na Li, Xiuhuan Wang, Wenjin Chen, Zhaofan Liu, Jiao Fang, Haitao Wen, Xiujuan Feng, Chunni Heng, Qingqing Zhang, Meifang Wang, Yan Yan

**Affiliations:** 1 Shaanxi Provincial People’s Hospital, Xi’an, 710032 China; 2 School of Basic Medicine, Inner Mongolia Medical University, Hohhot, 010110 China; 3 Peking University HuiLongGuan Clinical Medical School, Beijing, 100096 China; 4 College of Nursing and Rehabilitation, Xi’an Jiaotong University City College, Xi’an, 710018 China; 5 Tangdu Hospital, Fourth Military Medical University, Xian, 710038 China

**Keywords:** Perceived stress, Students, Nursing, Self-directed learning, Psychological capital

## Abstract

**Background:**

As indispensable reserves for the nursing workforce, undergraduate nursing students must possess self-directed learning abilities to consistently update their professional knowledge and adapt to the evolving demands of professional development. The acquisition of self-directed learning abilities can help undergraduate nursing students augment their theoretical knowledge and refine their clinical practice skills, thus fulfilling the demand from patients for high-quality nursing services. Hence, comprehending and investigating the factors that influence the development of self-directed learning abilities in nursing students is of paramount importance for nursing education and advancement of the nursing profession.

**Objectives:**

This study investigated the status of and associations between perceived stress, psychological capital, and self-directed learning abilities among undergraduate nursing students. Additionally, it examines the mediating role of psychological capital in the relationship between perceived stress and self-directed learning abilities. Thus, aiming to provide nursing educators with new directions for enhancing self-directed learning abilities.

**Design:**

A cross-sectional descriptive study.

**Methods:**

In February and March 2023, 900 undergraduate nursing students from 10 nursing schools completed an online questionnaire. The questionnaire included measures of perceived stress, psychological capital, and self-directed learning ability. Data were analyzed using SPSS 27.0 and the PROCESS macro tool.

**Results:**

The scores for perceived stress, psychological capital, and self-directed learning ability among undergraduate nursing students were 40.07 ± 5.90, 99.89 ± 16.59, and 87.12 ± 9.20, respectively. Self-directed learning abilities were negatively correlated with perceived stress (*r* = -0.415, *p* < 0.001) and positively correlated with psychological capital (*r* = 0.465, *p* < 0.001). Perceived stress was negatively correlated with psychological capital (*r* = -0.630, *p* < 0.001). Psychological capital partially mediated the relationship between perceived stress and self-directed learning abilities among undergraduate nursing students, with a mediation effect of -0.166, accounting for 49.55% of the total effect.

**Conclusion:**

This study found that undergraduate nursing students perceived high levels of stress, possessed low levels of psychological capital, and had moderate levels of self-directed learning. Perceived stress and psychological capital directly influenced undergraduate nursing students’ self-directed learning abilities, and perceived stress indirectly affected self-directed learning abilities through psychological capital. Nursing managers and educators should alleviate the perceived stress of undergraduate nursing students and cultivate their positive psychological capital to enhance self-directed learning abilities.

## Introduction

Self-directed learning ability is crucial for nursing students and nurses to engage in lifelong learning [1]. It involves proactive knowledge acquisition, application, independent problem-solving, and questioning [1, 2]. Undergraduate nursing students are an important reserve in the nursing workforce. Enhancing nursing students’ self-directed learning abilities is beneficial to professional development and knowledge updates for themselves and could help them meet patients’ demand for high-quality nursing care services. The self-directed learning abilities of undergraduate nursing students are closely associated with their academic success, learning interest, problem-solving ability, individual confidence, clinical practice abilities, and nursing professional values [1, 3, 4]. Recent studies have revealed that 51.97% of students attained academic success, suggesting that their performance was relatively low [5]. The academic success of nursing students can have implications for nursing shortages, academic funds, academic reputation, and training costs [6, 7]. Chen et al. [8] found that nursing students had low self-directed learning abilities. Meanwhile, perceived stress plays a vital role in the decline of self-directed learning abilities [9].

### Influence of perceived stress on self-directed learning ability

Perceived stress encompasses an individual's evaluation of stress levels arising from stimulating events, manifested as feelings of tension or lack of control [10]. A survey involving 1,010 participants revealed that 72% of students experienced high levels of stress, with 73% experiencing anxiety, depression, or other mental health problems [11]. According to the "general stress" theory, stress can contribute to maladaptive behaviors [12]. Students experiencing higher levels of perceived stress often exhibit lower self-control capabilities, more pronounced burnout, and a greater propensity to cope with stress through maladaptive behaviors (e.g., excessive internet or smartphone usage), potentially leading to a decline in their self-directed learning abilities [9, 13, 14]. Despite the established association between stress and self-directed learning among nursing students, the potential mechanism linking perceived stress and self-directed learning remains poorly understood.

### Psychological capital as potential mediating factor

Psychological capital is a positive psychological state manifested by individuals in the process of growth and development, including self-efficacy, hope, optimism, and resilience, which play important roles in individual adaptation to the environment and emotion regulation [15]. According to social cognitive theory, self-efficacy is an important factor that influences students' self-directed learning abilities. An individual's self-efficacy determines the motivation level and goals of later learning behaviors [16]. Students with higher self-efficacy are more likely to use self-directed learning strategies than those with lower self-efficacy, often demonstrating higher self-directed learning abilities [17].

Self-efficacy, hope, optimism, and resilience are essential constituents of psychological capital and closely correlated with the capacity for self-directed learning [18–20]. Individuals can sustain a constructive mind-set and employ proactive coping strategies when faced with adversity, thereby alleviating the detrimental consequences of stress. Stressors, such as academic, employment, further education, and life-related issues, can deplete individuals' limited psychological resources, leading to learning burnout and a decline in self-directed learning ability [9, 13, 14, 21, 22]. Perceived stress is an important predictive factor of psychological capital, which is a protective factor for self-directed learning ability [23, 24].

### Study purpose

Based on previous theoretical perspectives and empirical evidence that perceived stress is negatively associated with psychological capital and self-directed learning ability, psychological capital is positively associated with self-directed learning ability. However, the pathways of influence of these three variables remain largely unexplored in the current literature. Nurturing the development of self-directed learning abilities among undergraduate nursing students has consistently been a primary objective of nursing educators. Consequently, the primary focus of this study was on undergraduate nursing students, utilizing perceived stress as an independent variable, psychological capital as the mediating variable, and self-directed learning ability as the dependent variable. This study aimed to investigate the association between perceived stress and self-directed learning ability and to elucidate the mediating role of psychological capital. This study sought to provide nursing educators with a robust theoretical framework that enhances the self-directed learning abilities of undergraduate nursing students. Building on existing relevant research, this study formulates the following hypotheses: 1) Perceived stress exerts a negative predictive effect on self-directed learning ability among undergraduate nursing students, and 2) Psychological capital serves as a mediating factor in the relationship between perceived stress and self-directed learning ability among undergraduate nursing students.

### Theoretical framework: psychological capital may play a mediating role

Luthans formulated the notion of psychological capital in the context of positive organizational behavior and positive psychology [15]. Psychological capital is a vital positive personal resource that encompasses self-efficacy, hope, optimism, and resilience [25]. Specifically, individuals with elevated levels of psychological capital possess supplementary resources to manage their work and learning. They hold positive expectations of favorable outcomes, exhibit rapid recovery from setbacks, and display heightened optimism, even under adverse circumstances [15]. Individuals exhibiting elevated levels of psychological capital are inclined to perceive stress as a challenge rather than a threat, and possess enhanced coping abilities and regulatory strategies to manage and contend with stress. Consequently, this positive perceived stress contributes to sustaining a positive psychological state and fostering their motivation to learn, thereby augmenting their capacity for self-directed learning.

## Method

### Design and participants

A cross-sectional survey was conducted using convenience sampling to select undergraduate nursing students from 10 universities in Xi'an, Shaanxi Province, China, between February and March 2023. The inclusion criteria were as follows: (1) full-time undergraduate nursing students in a four-year program; (2) nursing undergraduate students who voluntarily signed an informed consent form. Students who were not enrolled in school for reasons, such as taking a leave of absence or joining the military, were excluded. The sample size was calculated with the formula *N* = 4Uα^2^S^2^/δ^2^ [26]. S = 0.68 was determined based on the preliminary experimental results. The allowable error δ is set to 0.1, and α is set to 0.05; therefore, *N* = 4 × 1.96^2^ × 0.68^2^/0.1^2^≈710. Considering the 15% invalid questionnaire rate, I have decided to set the minimum sample size as 817. A total of 900 undergraduate nursing students were included in this study—54 male students and 846 female students.

### Procedure

Following unified training, the questionnaire was distributed to eligible undergraduate nursing students using the Questionnaire Star platform. Quality control was as follows: each IP address was restricted to a single submission to prevent duplicate responses; the questionnaire completion process lasted approximately 15–20 min; invalid questionnaires were excluded, including those with missing responses, consistent answers to all items, or response times of less than 3 min. A total of 913 questionnaires were collected, 900 of which were valid after quality control, for a valid response rate of 98.58%.

### Ethical considerations

The present study complied with the Declaration of Helsinki and was approved by the Ethics Committee of Tangdu Hospital (Approval ID: TDLL-202210–17). All participants fully understood the study and voluntarily signed an informed consent form.

### Measurements

#### Demographic characteristics

Demographic characteristics included gender, grade, only child in the family, type of residence, family finances, and extracurricular activities, such as nursing profession, academic atmosphere, and academic achievement.

#### Perceived Stress Scale (PSS)

The perceived stress of undergraduate nursing students was measured using the PSS, originally compiled by Cohen et al. and revised by Yang et al. [10, 27]. The scale consists of 14 items in two dimensions: sense of losing control (7 items) and sense of tension (7 items). Each item was rated on a 5-point Likert scale ranging from 1 (never) to 5 (always). The tension dimension was scored from 7 to 35, with higher scores indicating greater tension. The loss of control dimension is scored from 7 to 35, with higher scores indicating a greater loss of control. The perceived stress score was the sum of the scores from these two dimensions, ranging from 14 to 70, with higher scores indicating higher stress perception. This scale has shown good reliability and validity in previous studies [28]. In this study, the overall Cronbach’s α was 0.74, and the Cronbach’s α of the tension dimension and loss of control dimension were 0.81 and 0.87, respectively.

#### Positive Psychological Capital Questionnaire (PPCQ)

The psychological capital of undergraduate nursing students was measured using the PPCQ developed by Zhang et al. [29] based on the scale developed by Luthans et al. [15]. The questionnaire consisted of 26 items, including self-efficacy (7 items), hope (6 items), optimism (6 items), and resilience (7 items). Each item is rated on a 7-point Likert scale ranging from 1 (completely disagree) to 7 (completely agree). The scores for self-efficacy, resilience, hope, and optimism dimensions ranged from 7 to 49, 7 to 49, 6 to 42, and 6 to 42, respectively. The total scores in each dimension represented the psychological capital score, ranging from 26 to 182, with higher scores indicating a higher level of psychological capital. This scale has demonstrated good reliability and validity in previous studies [30]. In this study, the overall Cronbach’s α was 0.81, and the Cronbach’s α of self-efficacy, resilience, hope, and optimism were 0.88, 0.70, 0.85, and 0.88, respectively.

#### Self-directed Learning Ability Scale (SLAS)

The self-directed learning ability of undergraduate nursing students was measured using the SLAS developed by Lin et al. [31]. The scale consists of three dimensions: self-management ability (10 items), information ability (11 items), and collaborative learning ability (7 items), with a total of 28 items. Each item is rated on a 5-point Likert scale ranging from 1 (completely matches) to 5 (completely does not match). The scores for self-management, information, and collaborative learning ability dimensions ranged from 10 to 50, 11 to 55, and 7 to 35, respectively. The total scores in each dimension represent the scores for self-directed learning ability, ranging from 28 to 140. Higher scores indicated higher levels of self-directed learning ability. This scale has shown good reliability and validity in previous studies [31]. In this study, the overall Cronbach’s α was 0.81, and the Cronbach’s α of self-management ability, information ability, and collaborative learning ability were 0.53, 0.67, and 0.52, respectively.

### Statistical methods

Statistical analysis was performed using SPSS 27.0, and PROCESS Macro. The Gaussian distribution of the data was assessed using the Kolmogorov–Smirnov (K-S) single-sample test and a P-P plot. The data were normally distributed. Descriptive statistics, including frequency, percentage, mean, and standard deviation were used to analyze participants' general demographic characteristics and their scores on perceived stress, psychological capital, and self-directed learning abilities. Independent t-tests and a one-way analysis of variance (ANOVA) were used to compare variations in self-directed learning abilities among participants based on their general demographic characteristics. Pearson’s correlation analysis was performed to investigate the associations between the three variables: perceived stress, psychological capital, and self-directed learning abilities. Harman's single-factor test was employed to assess common method bias arising from self-reported data [32]. PROCESS Model 4 was employed to explore the mediating role of psychological capital between perceived stress and self-directed learning abilities among undergraduate nursing students while controlling for all statistically significant covariates identified in the general demographic analysis [33]. This method is based on ordinary least squares regression and bootstrapping. Furthermore, to assess the impact of perceived stress on self-directed learning abilities among undergraduate nursing students, a bias-corrected percentile bootstrap distribution with a 95% confidence interval was calculated based on 5,000 bootstrap samples [33]. Statistical significance was set at a *p*-value < 0.05.

## Results

### Common method bias tests

Data in this study were collected through online questionnaire completion using the “QuestionStar” platform, which has the potential to introduce common method bias. To mitigate this bias, anonymous responses and reverse scoring techniques were implemented during data collection. Furthermore, the Harman single-factor test was administered to all the collected data, and an exploratory factor analysis was conducted on items measuring perceived stress, psychological capital, and self-directed learning abilities. Exploratory factor analysis identified 11 factors with eigenvalues > 1. The first factor accounted for only 26.80% of the total variance, which was significantly below the 40% threshold [32]. This suggests that the study did not exhibit severe common method bias [32].

### Descriptive characteristics and comparison of self-directed learning ability

This study involved 913 respondents, 900 of whom were undergraduate nursing students who completed the survey. This resulted in a high questionnaire completion rate of 98.58%. Within the cohort of 900 undergraduate nursing students, there were 38 male (6.38%) and 558 female students (93.62%). There were 195 (21.67%), 206 (22.89%), 260 (28.89%), and 239 (26.56%) participants in their first, second, third, and fourth years, respectively. The detailed general demographic characteristics are presented in Table 1.

**Table 1:** Demographic characteristics and comparison of self-directed learning ability among undergraduate nursing students (*N* = 900)

		SLA					
Variable	N(%)	Mean	SD	t / F	*p*	Post hop test	*p*
Gender				0.392	0.695		
Male	54(6.00)	87.59	9.85				
Female	846(94.00)	87.09	9.16				
Grade				0.827	0.479		
①First year	195(21.67)	86.64	9.36				
②Second year	206(22.89)	86.79	8.91				
③Third year	260(28.89)	87.02	8.85				
④Fourth year	239(26.56)	87.89	9.68				
Only child in family				0.301	0.763		
Yes	195(21.67)	87.29	9.89				
No	705(78.24)	87.07	9.01				
Type of Residence				87.48	9.94		
①Downtown	206(22.89)	87.48	9.94				
②Suburb	203(22.56)	86.35	8.97				
③Rural areas	491(54.56)	87.28	8.97				
Family finances				0.634	0.531		
①Low income	142(15.78)	86.42	10.63				
②Middle income	739(82.11)	87.22	8.87				
③High income	19(2.11)	88.37	10.53				
Extracurricular Activities				5.440	< 0.001		
Yes	536(59.56)	88.47	9.66				
No	364(40.44)	85.12	8.08				
Likes nursing profession				37.491	< 0.001		
①Like	247(27.44)	91.02	10.61			① > ②	< 0.001
②Generally like	589(65.44)	85.98	7.94			① > ③	< 0.001
③Dislike	64(7.12)	82.52	9.31			② > ③	0.003
Academic atmosphere				25.332	< 0.001		
①Intense atmosphere	229(25.44)	90.56	10.98			① > ②	< 0.001
②Moderate atmosphere	646(71.78)	86.10	8.11			① > ③	< 0.001
③Weak atmosphere	25(2.78)	81.88	9.47			② > ③	0.021
Academic achievement				20.785	< 0.001		
①Low achievers	49(5.44)	82.43	9.52			③ > ①	< 0.001
②Middle achievers	749(83.22)	86.79	8.47			③ > ②	< 0.001
③High achievers	102(11.34)	91.77	12.07			② > ①	0.001

Significant *p*-value are bold

*N* Number, *SLA* Self-directed learning ability, *SD* Standard deviation

The comparison of self-directed learning ability was compared among various general demographic variables using appropriate statistical analyses, including independent sample t-tests and analysis of variance (ANOVA). Regarding the self-directed learning ability of undergraduate nursing students, statistically significant differences were observed concerning their participation in extracurricular activities, affinity towards the nursing profession, academic atmosphere, and academic achievement (*p* < 0.001).

### Descriptive analysis of perceived stress, psychological capital, and self-directed learning ability

Table 2 displays the scores of the undergraduate nursing students in terms of perceived stress, psychological capital, and self-directed learning ability across all dimensions. The scores for perceived stress, psychological capital, and self-directed learning ability among nursing undergraduate students were 40.07 ± 5.90, 99.89 ± 16.59, and 87.12 ± 9.20, respectively.

**Table 2:** Descriptive statistics of all measures used in the current study (*N* = 900)

	Minimum	Maximum	Mean	SD	Skewness	Kurtosis
Perceived stress	14.00	70.00	40.07	5.90	0.08	0.16
Sense of loss of control	7.00	35.00	21.91	4.76	0.08	0.16
Sense of tension	7.00	35.00	18.16	4.10	0.08	0.16
Psychological capital	26.00	182.00	99.89	16.59	0.08	0.16
Self-efficacy	7.00	49.00	25.64	4.87	0.08	0.16
Hope	6.00	42.00	24.21	4.77	0.08	0.16
Resilience	6.00	42.00	25.83	4.46	0.08	0.16
Optimism	7.00	49.00	24.22	5.04	0.08	0.16
Self-directed learning ability	28.00	140.00	87.12	9.20	0.08	0.16
Self-management ability	10.00	50.00	31.16	3.41	0.08	0.16
Information literacy	11.00	55.00	34.43	4.50	0.08	0.16
Collaborative learning ability	7.00	35.00	21.53	2.90	0.08	0.16

The Perceived Stress Scale ranges from 14 to 70, with higher scores indicating higher levels of perceived stress: 14 to 28 indicate low levels, 29 to 42 indicate moderate levels, 43 to 56 indicate high levels, and 57 to 70 indicate extremely high levels of perceived stress. The score range of Psychological Capital is from 26 to 182, and higher scores indicate higher levels of Psychological Capital. The score range of the Self-Directed Learning Ability Scale is from 28 to 140, and higher scores indicate higher levels of self-directed learning ability

*SD* Standard deviation

### Correlations of the study variables

The correlation coefficients between the variables in this study are listed in Table 3. The results of the Pearson correlation analysis showed a negative correlation between self-directed learning ability and perceived stress (*r* = -0.415, *p* < 0.001), a positive correlation between self-directed learning ability and psychological capital (*r* = 0.465, *p* < 0.001), and a negative correlation between perceived stress and psychological capital (*r* = -0.630, *p* < 0.001).

**Table 3:** Correlations of the study variables (*N* = 900)

Variables	1	2	3	4	5	6	7	8	9	10	11	12
1.Self-directed learning ability	1											
2.Self-management ability	0.820^***^	1										
3.Information literacy	0.901^***^	0.585^***^	1									
4.Collaborative learning ability	0.810^***^	0.519^***^	0.619^***^	1								
5.Psychological capital	0.465^***^	0.379^***^	0.393^***^	0.419^***^	1							
6.Self-efficacy	0.384^***^	0.301^***^	0.322^***^	0.364^***^	0.882^***^	1						
7.Hope	0.443^***^	0.398^***^	0.359^***^	0.379^***^	0.894^***^	0.689^***^	1					
8.Resilience	0.420^***^	0.322^***^	0.374^***^	0.374^***^	0.801^***^	0.664^***^	0.595^***^	1				
9.Optimism	0.369^***^	0.298^***^	0.312^***^	0.337^***^	0.888^***^	0.701^***^	0.805^***^	0.548^***^	1			
10.Perceived stress	0.415^***^	0.352^***^	0.344^***^	0.369^***^	0.630^***^	-0.573	0.526^***^	0.625^***^	0.473^***^	1		
11. Sense of loss of control	0.301^***^	0.256^***^	0.238^***^	0.286^***^	0.499^***^	0.459^***^	0.418^***^	0.433^***^	0.421^***^	0.724^***^	1	
12. Sense of tension	0.248^***^	0.209^***^	0.219^***^	0.200^***^	0.329^***^	0.292^***^	0.272^***^	0.397^***^	0.192^***^	0.600^***^	0.118^***^	1

^***^ *p* < 0.001

### Mediation effect test

Following the standardization of all variables in this study and control of all statistically significant covariates identified in the general demographic analysis, we employed Model 4 from the SPSS macro program developed by Hayes to examine the mediating role of psychological capital in the relationship between perceived stress and self-directed learning ability [33]. The findings of this study indicated that the regression equation was statistically significant (*R* ^*2*^ = 0.225, *F* = 51.810, *p* < 0.001). Regression analysis showed that perceived stress negatively predicted psychological capital (*β* = -0.573, *SE* = 0.027, *t* = -21.229, *p* < 0 0.001), negatively predicted self-directed learning ability (*β* = -0.168, *SE* = 0.037, *t* = -4.532, *p* < 0.001), while psychological capital significantly positively predicted self-directed learning ability (*β* = 0.291, *SE* = 0.038, *t* = 7.750, *p* < 0.001).

**Fig. 1:**
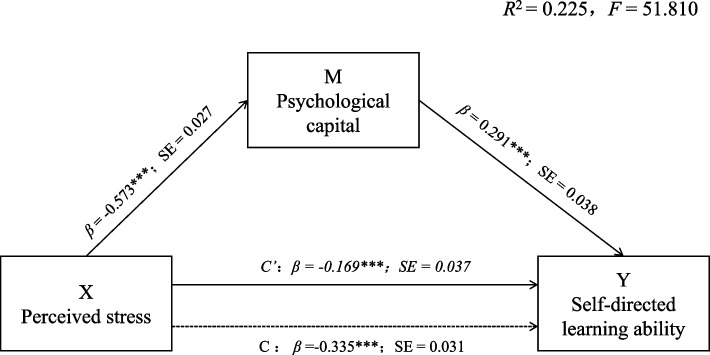
The mediation of psychological capital in the relationship between perceived stress and self-directed learning ability with standardized beta values and standard error. Notes: *** *p* < 0.001

The mediation model was examined using the Hayes’ methodology. Bootstrap with 5,000 iterations was used to calculate the 95% confidence intervals [33]. The results of this analysis are presented in Table 4. The direct impact of perceived stress on self-directed learning ability is estimated to be -0.168, accompanied by a mediation effect ratio of 50.45%. The 95% confidence interval for the mediation effect ranges from -0.242 to -0.096, wherein zero was not encompassed, signifying a statistically significant direct effect. The mediated impact of psychological capital on the association between perceived stress and self-directed learning ability is estimated to be -0.166, accompanied by a mediation effect ratio of 49.55%. The 95% confidence interval for the mediation effect ranges from -0.232 to -0.106, wherein zero was not encompassed, indicating a significant mediating effect of psychological capital. Figure 1 illustrates the specific pathways through which perceived stress influences self-directed learning ability among undergraduate nursing students.

**Table 4:** Total, direct, and indirect effects of perceived stress on professional identity (*N* = 900)

Path	Effect	Boot SE	Boot LLCI	Boot ULCI	Relative Mediation Effect
Total effect of X on Y	-0.335	0.031	-0.397	-0.274	
Direct effect of X on Y	-0.169	0.037	-0.242	-0.096	50.45%
Indirect effects 1 (X → M → Y)	-0.166	0.032	-0.232	-0.106	49.55%

Boot LLCI the lower limit confidence interval of effects estimated by Bootstrap Method, Boot SE the standard error of effects estimated by Bootstrap method, Boot ULCI the upper limit confidence interval of effects estimated by Bootstrap method. X = Perceived stress; M = Psychological capital; Y = Self-directed learning ability

## Discussion

This study investigated the potential mediating role of psychological capital between perceived stress and self-directed learning ability. The results demonstrated a significant negative predictive relationship between perceived stress and self-directed learning ability among undergraduate nursing students, with psychological capital as a mediating factor. These findings suggest that among undergraduate nursing students, the higher the perceived stress, the lower the psychological capital, which further leads to a decrease in their self-directed learning ability.

### Direct effect of perceived stress on self-directed learning ability

The findings revealed that perceived stress was a risk factor for the self-directed learning ability of undergraduate nursing students. This validates Hypothesis 1 and is consistent with previous research [9, 13, 14]. Stress is widely recognized as pervasive throughout the entire process of undergraduate nursing education [34]. Undergraduate nursing students encounter a multitude of pressures during their academic journey, including academic and social pressure, interpersonal and romantic relationships, the pursuit of further education, employment, and the transition from student to clinical nurse. The amalgamation of these multiple tasks imposes substantial stress on students. Moderate stress can facilitate active learning, consequently bolstering self-directed learning capabilities. Conversely, excessive stress can lead to behaviors that undermine mental and physical well-being, including social anxiety, loneliness, academic burnout, and smartphone addiction, consequently diminishing self-directed learning ability [1, 13, 14, 24].

Moreover, these findings substantiate the general stress theory, which postulates a substantial influence of stress on undergraduate nursing students [12]. Individuals who perceive higher stress levels are more likely to employ maladaptive coping strategies to alleviate stress, hindering the development of self-directed learning abilities. Conversely, individuals with lower stress perception experience less depletion of self-regulatory resources, which promotes the cultivation of self-directed learning abilities [9, 13, 14, 24].

### Mediating mechanism of psychological capital

The main results indicated a significant mediating effect of psychological capital between perceived stress and self-directed learning ability, confirming Hypothesis 2. This suggests that undergraduate nursing students with higher levels of perceived stress experience increased depletion of psychological capital, consequently leading to a decline in their self-directed learning ability. Perceived stress negatively predicts psychological capital; individuals with higher stress levels are more likely to deplete their psychological resources, which is consistent with previous research [23]. Psychological capital, which serves as a positive psychological resource for individuals, can effectively mitigate the adverse impact of stress on oneself [35].

Psychological capital can positively predict individuals' self-directed learning ability [23, 24]. Self-efficacy, optimism, hope, and resilience are integral elements of psychological capital that play a vital role in fostering self-directed learning ability [18–20, 32]. Self-efficacy can stimulate innate motivation and unwavering determination, thereby motivating active involvement in self-directed learning. Hope serves as an inspiration for individuals to pursue goal attainment relentlessly through devoted learning endeavors, thereby augmenting their self-directed learning abilities. Optimism empowers individuals with the capacity to effectively manage and resolve challenges, contributing to surmounting learning obstacles and enhancing self-directed learning ability. Resilience provides valuable assistance for individuals in confronting academic challenges, surmounting obstacles, and ultimately enhancing their self-directed learning abilities.

Furthermore, the findings of this study validate the stress-coping model, indicating that individuals' psychological capital can influence their perception and coping strategies [36]. Undergraduate nursing students with higher levels of psychological capital tend to view stress as a challenge rather than a threat. They frequently exhibit superior coping skills and regulatory strategies that enable them to manage and address stress effectively, leading to enhancements in their self-directed learning abilities. Perceived stress has the potential to affect self-directed learning through the depletion of psychological capital. Consequently, self-directed learning abilities can be enhanced by reducing perceived stress and enhancing psychological capital.

### The level of perceived stress

The present study's findings indicate that perceived stress levels among undergraduate nursing students are elevated compared to the results reported by Onieva-Zafra et al. [37] and Xu et al. [38]. One possible explanation for this phenomenon is that undergraduate nursing students, as a distinct group within a university, often have more academic and professional responsibilities than students in other fields, leading to higher levels of stress. Extensive research has indicated that severe stress experienced during medical education can exert myriad detrimental impacts on individuals, encompassing depression, anxiety, insomnia, internet addiction, feelings of isolation, and, in extreme cases, suicidal tendencies [14, 39]. One study revealed that a substantial majority (74.73%) of nursing students advocated the integration of psychological capital education into the medical curriculum [40]. Hence, it is imperative that nursing administrators and educators monitor the mental well-being of nursing students and consistently implement group-based psychological interventions.

### The level of psychological capital

The results of this study revealed that the level of psychological capital among undergraduate nursing students was markedly lower than that reported in the study conducted by Wang et al. [22]. Individuals with elevated psychological capital are adept at managing academic pressure by utilizing internal resources, cultivating optimism, and bolstering self-efficacy. These abilities help them cope effectively with stress, overcome obstacles, avoid burnout, and maintain dedication to professional growth. Research conducted by Dello et al. [41] has consistently shown that training interventions focused on psychological capital can lead to significant improvements in individuals' levels of psychological capital, resulting in long-lasting effects. Considering the relatively modest level of psychological capital, substantive opportunities for advancement have persisted. Consequently, fostering and augmenting the psychological capital of undergraduate nursing students has emerged as an exigent imperative.

### The level of self-directed learning ability

The findings of this study suggest that the scores of undergraduate nursing students concerning autonomous learning ability were markedly lower than those reported in the study conducted by Tang et al. [42], yet they still surpassed the average level of self-directed learning ability in China [43]. These findings suggest that the self-directed learning ability of this specific group of undergraduate nursing students was moderately positioned, indicating significant potential for enhancement. Within the context of this study, the scores pertaining to self-management and information processing abilities were relatively high in relation to the domain of autonomous learning ability, whereas the score for collaborative learning ability was comparatively low. Supporting research has indicated that innovative teaching approaches such as group presentations and flipped classrooms have the potential to enhance students' ability to work effectively in teams [44]. Thus, it is imperative for nursing educators to integrate conventional and innovative educational approaches to foster intrinsic motivation, consequently bolstering the self-directed learning abilities of undergraduate nursing students.

## Implications for research and practice

The findings of this study may be helpful in enhancing the self-directed learning abilities of undergraduate nursing students. These results theoretically emphasize the importance of considering psychological capital as a potential mediating factor in understanding the impact of perceived stress on self-directed learning ability. To improve the level of self-directed learning ability, the following recommendations are made. First, nursing educators have the potential to enhance self-directed learning by providing effective stress management techniques and strategies. Educators can foster students' learning motivation by nurturing psychological capital. Second, students must acknowledge the intricate association between perceived stress and self-directed learning. The proactive pursuit of psychological support and learning resources can significantly facilitate students’ effective stress management and enhance their learning outcomes. Finally, nursing managers should regularly arrange for psychological experts to provide instructions. Guiding students to evaluate daily life stressors objectively from a positive perspective is a potent means of effectively attenuating the impact of perceived stress on self-directed learning ability, thereby facilitating the cultivation of self-directed learning.

### Limitations

This study has certain limitations. First, 94% of the participants were female, and 6% were male. Future studies should include a larger proportion of male participants to obtain more comprehensive and representative results. Second, despite efforts to ensure stringent control over the survey process, it is crucial to acknowledge that the self-administered questionnaire format and inherent subjectivity of psychological assessments may create potential biases in self-reporting. Consequently, the conclusions derived from this investigation can only provide a descriptive depiction of the present circumstances, with inherent limitations on their generalizability. Finally, the use of a cross-sectional research design in this study limited the ability to establish causal relationships in the research findings, offering only suggestive evidence. Future research should employ longitudinal designs or experimental approaches to investigate the causal relationship between perceived stress and self-directed learning ability. Previous research has demonstrated that the utilization of Online Photovoice (OPV), Online Interpretative Phenomenological Analysis (OIPA), and Community-Based Participatory Research (CBPR) approaches can assess the cognitive, affective, and behavioral dimensions of individuals [45–48]. Subsequent studies should employ OPV and CBPR methodologies to investigate the influence of psychological capital on perceived stress and self-directed learning ability among undergraduate nursing students.

## Conclusion

The findings highlight the experiences of undergraduate nursing students and their elevated levels of perceived stress, limited psychological capital, and moderate self-directed learning ability. This study examined the mediating role of psychological capital in the relationship between perceived stress and self-directed learning ability among undergraduate nursing students. The research findings provided evidence of a significant correlation between heightened levels of perceived stress and diminished psychological capital, consequently affecting the decline in self-directed learning abilities. Therefore, mitigating perceived stress and bolstering psychological capital can effectively enhance the self-directed learning abilities of undergraduate nursing students.

## Data Availability

De identified textual data are available on reason request from the "Corresponding author".

## Abbreviations

PSS: Perceived Stress Scale
PPCQ: Positive Psychological Capital Questionnaire
SLAS: Self-Directed Learning Scale
